# Infant burn injuries related to water heating for powdered infant formula preparation

**DOI:** 10.3389/fped.2023.1125112

**Published:** 2023-05-04

**Authors:** Katelyn V. Chiang, Erica H. Anstey, Steven A. Abrams, Cria G. Perrine

**Affiliations:** ^1^ Division of Nutrition, Physical Activity, and Obesity, National Center for Chronic Disease Prevention and Health Promotion, Centers for Disease Control and Prevention, Atlanta, GA, United States; ^2^ McKing Consulting Corporation, Atlanta, GA, United States; ^3^ Department of Pediatrics, Dell Medical School, University of Texas at Austin, Austin, TX, United States; ^4^ Commissioned Corps of the U.S. Public Health Service, Rockville, MD, United States

**Keywords:** infant formula, infant nutrition, burns, injuries, nutrition guidelines

## Abstract

**Background:**

Guidance for preparing powdered infant formula (PIF) helps to ensure it meets the nutritional needs of infants and is safe to consume. Among safety concerns is *Cronobacter sakazakii* contamination which can lead to serious infections and death. PIF preparation guidance varies; there is a lack of consensus on whether there is a need to boil water to inactivate potential *Cronobacter* and for how long to let the water cool before reconstitution. We sought to quantify the burden of burn injuries among infants related to water heating for PIF preparation. Estimating this burden may help inform preparation recommendations.

**Methods:**

Burn injuries among infants <18 months of age were identified from 2017 to 2019 National Electronic Injury Surveillance System data collected from sampled hospital emergency departments. Injuries were classified as related to PIF water heating, potentially related to PIF water heating but with undetermined causation, related to other infant feeding aspects, or unrelated to infant formula or breast milk feeding. Unweighted case counts for each injury classification were determined.

**Results:**

Across sampled emergency departments, 7 PIF water heating injuries were seen among the 44,395 injuries reported for infants <18 months. No reported PIF water heating injuries were fatal, but 3 required hospitalization. Another 238 injuries potentially related to PIF water heating but with undetermined causation were also seen.

**Conclusion:**

Preparation guidance should consider both the potential risk for *Cronobacter* infection and the potential risk for burns.

## Introduction

Although breast milk is the optimal source of infant nutrition (1), most infants in the United States receive infant formula at some point during their first year of life. Just 1 in 4 US infants born in 2019 were exclusively breastfed through 6 months, as recommended (1, 2). Guidance for preparing powdered infant formula (PIF) helps to ensure the formula meets the nutritional needs of infants and is safe to consume. Safety concerns include the use of a safe water source to reconstitute PIF and potential contamination of formula from the gram-negative bacteria *Cronobacter sakazakii*, formerly known as Enterobacter sakazakii, which can result from both environmental exposure (i.e., unclean bottles and surfaces) and intrinsically contaminated PIF (3–5). Since 2014, US manufacturers have been required to test PIF for *Cronobacter* before further distribution (6). Following this testing mandate, from 2015 to 2018, 2–4 cases of infant *Cronobacter* infection were reported each year (7). More recently, in 2022, following the investigation of 2 infant *Cronobacter* cases, a voluntary recall of powdered infant formula led to supply chain issues and a nationwide infant formula shortage, which brought more public attention to the safety of PIF and preparation methods (8, 9).

In 2006, the World Health Organization (WHO) released guidelines on the safe preparation, storage, and handing of PIF following a series of *Cronobacter* infection outbreaks in neonatal intensive care units (10–12). These guidelines for reconstituting PIF instruct parents and home-based caregivers to clean and disinfect surfaces and hands, bring water to a rolling boil, pour water that has been cooled to no less than 70°C/158°F (cool for no more than 30 min) into a clean and sterilized bottle, add PIF to the bottle and mix, and then further cool the reconstituted PIF to a safe feeding temperature before serving (12). PIF preparation guidance has varied in the United States, and there has been a lack of consensus on whether there is a need to boil water and for how long to let the water cool before reconstituting PIF (13). Water may be boiled and cooled before mixing PIF, which is primarily to ensure a safe water source (3). Alternatively, water may be boiled and mixed with PIF while still hot (at least 70°C/158°F), which is intended to inactivate any potential *Cronobacter* (11, 12).

A 2019 commentary highlighted the variation in PIF preparation guidance and the lack of agreement surrounding water heating and water temperatures when reconstituting PIF (13). Considering more than 300 children are estimated to be treated in emergency departments (EDs) each day for burn-related injuries (14), it is important to consider the potential risk of burns from boiling water for PIF preparation along with the potential risk of *Cronobacter* infection (13).

In this cross-sectional study, we sought to better quantify the burden of burn injuries among infants related to water heating for PIF preparation (hereafter referred to as PIF water heating). Estimating the burden of burn injuries related to PIF water heating may help inform discussions related to the risks and benefits of water heating for PIF preparation and wider PIF preparation recommendations.

## Materials and methods

The National Electronic Injury Surveillance System (NEISS) collects data on consumer product-related injuries from a probability sample of approximately 100 hospital EDs representing more than 5,000 hospitals with EDs across the United States and US territories. Trained NEISS coordinators in each hospital abstract data from medical records of patients presenting to the ED with a consumer product-related injury, assign each case at least 1 product code from a standardized coding manual corresponding to the related consumer product, and provide a brief 1–2 line narrative describing the incident scenario (15–17).

**Figure 1:**
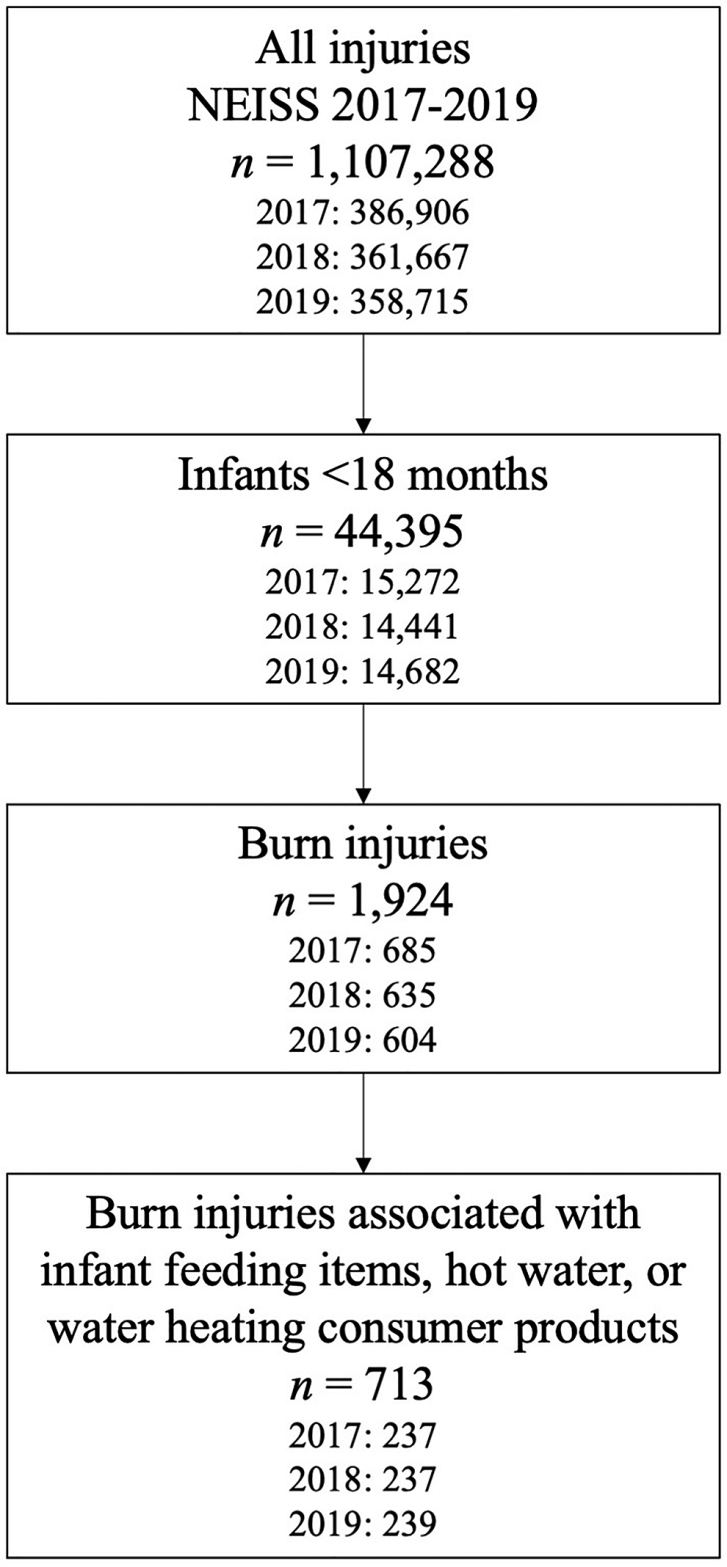
Identification of burn injuries associated with infant feeding items, hot water, and water heating consumer products for further review for relation to water heating during powdered infant formula preparation.

To identify cases for review, the authors first restricted 2017–2019 NEISS data to infants <18 months of age (*n* = 44,395) (Figure 1). Next, the authors queried the dataset for potential burn injuries (*n* = 1,924) using diagnosis codes 48 (burns, scald), 51 (burns, thermal), and 47 (burns, not specified). Injuries associated with diagnosis code 71 (other/not stated) were also included if their associated narratives referred to “burn” or “scald.” Beginning in 2019, injuries could have a second associated diagnosis code; for consistency, the authors examined only the primary diagnosis code across all years.

The authors then further restricted injuries to those associated with infant feeding items, hot water, and water heating consumer products (*n* = 713) by querying product codes 1509 (baby bottles or nipples), 1934 (hot water), 1510 (bottle warmers), 264 (microwave ovens), 278 (electric ranges or ovens), 279 (gas ranges or ovens), 280 (other ranges or ovens), 281 (ranges or ovens, not specified), 217 (electric coffee makers or teapots), 405 (unpowered coffee makers or teapots), 452 (coffee makers or teapots, not specified), 269 (electric kettles or hot pots), and 224 (hot plates) (Figure 1). Each injury could list up to 3 associated product codes; all 3 product code fields were queried. Because NEISS does not include injuries related to food products, queries specific to infant formula products were not possible (18). To determine each injury's association with PIF water heating, 2 authors (KC, EA) first reviewed a sample of injury narratives and collaboratively developed 14 injury classification codes based on likely causes of each injury. Classification codes included 1) PIF preparation; 2) hot water, use not specified; 3) hot surface used to heat water, use not specified; 4) bottle warming; and 5) infant feeding item sanitation. Injuries coded as “PIF preparation” (code 1) included narratives that described formula or bottle preparation with hot water; however, PIF reconstitution did not have to be explicitly mentioned for inclusion. Injuries coded as “hot water, use not specified” (code 2) or “hot surface used to heat water, use not specified” (code 3) involved hot water or water heating appliances or devices such as stoves and hotplates without descriptions of stated uses for the heated water. Injuries coded as “bottle warming” (code 4) had narratives similar but distinct from injuries coded as “PIF preparation” (code 1). Rather than descriptions of bottle preparation, narratives coded as “bottle warming” (code 4) included frequently used methods for bottle warming such as using bottle warming devices, heating prepared bottles in microwaves, or placing prepared bottles in hot water to warm the bottles' contents. Similarly, narratives of injuries coded as “infant feeding item sanitation” (code 5) included frequently used methods for bottle cleaning such as boiling bottle parts. Remaining classification codes (codes 6–14) included injuries determined to be unrelated to PIF water heating, such as those related to hot water used for bathing, or miscellaneous injuries unrelated to infant feeding (see Supplementary Material Table S1 for a complete description of all codes).

The two authors (KC, EA) then independently reviewed and coded each case, reaching a concordance of 91.2%. KC and EA then discussed discrepancies in coding (*n* = 63) and either came to a consensus (*n* = 59) or consulted a third author (CP) to determine the injury's classification (*n* = 4). KC, EA, and CP then further delineated injuries as those related to PIF water heating (code 1), those potentially related to PIF water heating but with undetermined causation (codes 2 and 3), those related to other infant feeding aspects (codes 4 and 5), and those unrelated to infant formula or breast milk feeding (codes 6–14) (Supplementary Material Table S1).

The authors determined unweighted case counts for each burn injury classification. Small case counts prevented the authors from further applying complex survey procedures to calculate stable national estimates such as weighted case counts and stable estimates of occurrence such as incidence rates. The authors also examined disposition of each PIF water heating case. This secondary data analysis of public use, de-identified data is not research involving human subjects and did not require Centers for Disease Control and Prevention (CDC) Institutional Review Board review.

## Results

Of the 713 burn injuries among infants <18 months of age associated with infant feeding items, hot water, or water heating consumer products in this 2017–2019 review, 442 injuries were determined to be unrelated to infant milk feeding. Most of these unrelated injuries were associated with bathing or food and beverage preparation other than infant formula or breast milk.

A total of 7 injuries related to PIF water heating were identified. Another 238 injuries potentially related to PIF water heating but with undetermined causation were also identified. Of these potential PIF water heating injuries, 188 were associated with contact with hot water, use not specified and 50 were associated with contact with hot surfaces used to heat water, use not specified, such as a stove or hot plate. A further 26 injuries related to other infant feeding aspects were also identified, including 24 associated with warming bottles and 2 associated with infant feeding item sanitation (Table 1).

**Table 1:** Burn injuries associated with infant feeding items, hot water, and water heating consumer products and relation to infant feeding—NEISS, 2017-2019.

	Count
**PIF water heating**	**7**
**Potentially related to PIF water heating**	**238**
Hot water, use not specified	188
Hot surface used to heat water, use not specified	50
**Related to other infant milk feeding aspects**	**26**
Bottle warming	24
Infant feeding item sanitation	2
**Unrelated to infant formula or breast milk feeding**	**442**

Powdered infant formula, PIF.

The 7 injuries directly related to PIF water heating ranged from 1 to 4 cases a year across 2017–2019 and occurred among infants aged 2–14 months. Among these cases, 4 were examined or treated and released or not admitted but transferred for treatment to another department of the same facility, 1 was treated and transferred to another hospital, and 2 were treated and admitted for hospitalization (Table 2). No injuries collected by NEISS related to PIF water heating resulted in fatalities.

**Table 2:** Burn injuries associated with PIF water heating—NEISS, 2017–2019.

Case	Hospitalization Status	Narrative
1	Examined or treated and released or not admitted but transferred for treatment to another department of the same facility	4MOM mom mixing formula whot (*sic*) water, spilled water on abdomen; burn injury of abdomen
2	Treated and transferred to another hospital	9MOM leg burn s/p boiling water spilled on Pt while moms *(sic)* boyfriend was preparing the baby a bottle. Dx 2nd degree burn of L leg
3	Examined or treated and released or not admitted but transferred for treatment to another department of the same facility	6MOF mom spilled hot water on Pt's abdomen, thigh when making a bottle 2 days ago; burn injury of abdomen, thigh
4	Examined or treated and released or not admitted but transferred for treatment to another department of the same facility	5MOM was spashed *(sic)* with scalding water while mom was preparing a bottle; 2nd degree burn of upper right arm
5	Examined or treated and released or not admitted but transferred for treatment to another department of the same facility	14MOF put Lt hand inside a cup of boiled water, per mom she was heating up water to make her a bottle and she reached into a hot water cup, burning her hand, family put *** *(sic)* on her hand. Dx blisters w/epidermal loss due to burn (second degree) to Lt hand
6	Treated and admitted for hospitalization	2MOM was burned on the left foot and knee when mom poured hot water from a *** *(sic)* into his bottle and it spilled/splashed. Dx: 2nd degree burn of knee and foot.
7	Treated and admitted for hospitalization	13MOM presented to ED after mother waste *(sic)* a pot of hot water on the baby while trying to pour to bottle. Dx: partial thickness burn of abdomen

Powdered infant formula, PIF; month old male, MOM; month old female, MOF; status post, s/p; patient, Pt; diagnosis, Dx; left, L, Lt; with, w/; emergency department, ED.

## Discussion

Although the true burden of burns related to water heating for PIF preparation is difficult to determine, these counts offer some insights. Over 3 years of data collection, 7 PIF water heating injuries were seen in EDs in NEISS hospitals across the United States. Although 3 reported injuries required hospitalization, none were fatal; however, fatalities may be underreported as NEISS is not a good source for fatal injuries. Another 238 injuries potentially related to PIF water heating but with undetermined causation were also seen in EDs, although many of these were likely not related to PIF preparation. These data demonstrate that some infants in the United States are burned during PIF preparation, and that, though uncommon, some of these injuries are serious enough for ED visits and sometimes further hospitalization.

Notably, this analysis may have underestimated the true burden of PIF water heating-related injuries. These counts likely underestimate PIF water heating-related injuries as only cases appearing in EDs are accounted for in NEISS data, thus less severe burns not requiring emergency medical attention were not captured. Further, the 1–4 PIF water heating injuries estimated to occur each year are drawn from unweighted case counts among a sample of approximately 100 hospitals out of more than 5,000 hospitals with EDs across the United States. Small case counts prevented further extrapolation of these 1–4 annual cases into stable nationally representative weighted estimates for PIF water heating injuries. However, combining the 7 cases related to PIF water heating and those 238 cases potentially related to PIF water heating but with undetermined causation yields stable weighted estimates for possible PIF water heating burn injuries across the 3-year study period. Using this methodology, an estimated 1,706 infants presented to EDs for burn injuries possibly related to PIF water heating in the United States from 2017 to 2019. The true burden of infant burns related to PIF water heating requiring emergency care and presenting to the ED between 2017 and 2019 lies between 7 and 1,706 cases, likely far towards the lower end as the authors' definition of possible cases was liberal. Additionally, parents, caregivers, and other household children could also have experienced burns related to PIF preparation that were not captured in this study.

A 2012 research study among parents in the United Kingdom suggested that although many parents were confident in their ability to prepare PIF safely, most were not aware PIF was not sterile before opened and found it difficult to judge water temperature when reconstituting PIF (19). Parents and caregivers may lack awareness of underlying reasons for water boiling and correct, safe methods for PIF preparation. Those they turn to for expertise may have disparate recommendations.

Consensus is lacking on when water boiling during PIF preparation is necessary, for whom water boiling is needed, and if water is boiled, at which temperature to reconstitute PIF. CDC does not recommend heating water universally when preparing PIF as recommended by WHO, but cautions that if infants are at increased risk of infection (those who are younger than 2 months, were born prematurely, or have weakened immune systems), parents and caregivers may consider taking extra precautions to protect against *Cronobacter* by boiling water and then letting it cool for about 5 minutes (water should be at least 70°C/158°F) before mixing with PIF and further cooling to body temperature before consumption (3, 4). Following the PIF recall related to the 2022 *Cronobacter* investigation, consensus around PIF preparation guidelines has increased among federal agencies and professional organizations, such as the American Academy of Pediatrics (20, 21). However, PIF preparation instructions from Special Supplemental Nutrition Program for Women, Infants, and Children (WIC) programs may vary from state to state (22–25). Similarly, PIF manufacturers also provide preparation instructions on their product containers and product webpages. Although their instructions warn that PIF is not a sterile product, manufacturers do not provide instructions on preparing PIF with hot water (at least 70°C/158°F) to destroy potential bacterial contamination such as *Cronobacter*. Rather, the manufacturers' instructions generally advise parents and caregivers to ask their baby's doctor if they should use water that has been boiled and then cooled when preparing PIF, with the intention to protect infants when the safety of the water source is uncertain (26–28).

These inconsistencies in guidelines might be resulting in confusion among parents, caregivers, and pediatricians concerning when water heating is needed. Water heating might be occurring when it is not needed, thus increasing risk of related burn injuries. Further research on parental and caregiver knowledge of PIF preparation recommendations and the extent to which recommendations are followed might be useful in identifying opportunities to prevent both *Cronobacter* infection and burn injuries.

Efforts to clarify existing recommendations and to offer additional guidance on how to safely put recommendations into practice might help to reduce burn injuries. Guidance does not always clearly differentiate between water heating to ensure a safe, clean water source, such as during emergencies (29), and water heating to inactivate *Cronobacter*, such as for infants at increased risk of infection (4). Highlighting both the rationale for precautions and recommended steps to take during PIF preparation in both scenarios might help increase understanding and reduce ineffective or unnecessary water heating. Factors such as the correct water temperature for reconstitution of PIF to inactivate *Cronobacter* and safe water temperature for feeding and how long it takes boiled water to cool to those temperatures could also be further explained and clarified. Additionally, pediatricians and others who provide guidance on infant feeding might offer further education on how to safely implement PIF preparation recommendations to avoid burn injuries.

Parent and caregiver education on general hot water safety might also help reduce PIF water heating-related burns among infants and other burns related to daily household tasks such as bathing and food and beverage preparation. Additional research on effective prevention of scalds in children may also be warranted (30). Further, increased education on bottle warming might also be needed. Warming bottles is not necessary, but if a parent or caregiver wishes to warm a bottle, they should avoid using a microwave (which can create hot spots that can cause scalds) and instead hold the bottle under running warm water (3). Bottle warming devices heat infant formula and breast milk to precise temperatures but may be cost-prohibitive.

Although this study utilized recent national data and the methodology used to identify cases was inclusive of many potentially related causes, findings from this study are subject to several limitations. First, exhaustive information was not available for each case as narratives were limited to only a few sentences. The authors may have misclassified an injury's association with PIF water heating. For example, injuries that were described as resulting from bottle preparation with hot water and were thus classified as being related to PIF preparation by the authors may have been truly caused by bottle warming but were described using preparation rather than warming phrasing. Additionally, as previously mentioned, case counts may be underestimated due to severity bias as only burns serious enough to prompt an ED visit were examined. However, to the authors' knowledge, national data for burns examined and treated in outpatient settings that include narrative incident scenarios allowing for identification of underlying cause are not available. Finally, because NEISS does not capture non-consumer product-related injuries, some burns related to PIF water heating may not have been captured in the data because infant formula is not classified as a consumer product by the Consumer Product Safety Commission. Since many other products used in PIF preparation (i.e., hot water, stoves, microwaves, and bottles) were queried, the impact on estimates would likely be minimal; however, cases that mentioned only infant formula with no other consumer product would not be captured in NEISS data.

Over the past 2 decades, the threat of *Cronobacter* infections resulting from contaminated infant formula has led to the convening of global expert meetings, the development of formula preparation guidelines, and the mandating of manufacturer testing requirements (6, 10, 11, 12, 31). More recently, both the potential contamination of PIF (8, 9) and the potential unintended consequences of increased risk of infant burns as a result of stringent PIF water heating recommendations have received attention (13). Increased parental and caregiver education on hot water safety by pediatricians and others who guide infant feeding may help to reduce these burn injuries. These estimates of the burden of burn injuries related to PIF water heating can be used to help inform discussions related to the risks and benefits of water heating for PIF preparation and wider PIF preparation recommendations. Guidance on PIF preparation should consider the potential for risk of burns to infants as a result of PIF water heating.

**Note:** The findings and conclusions in this report are those of the authors and do not necessarily represent the official position of the Centers for Disease Control and Prevention.

## Data Availability

Publicly available datasets were analyzed in this study. This data can be found here: https://www.cpsc.gov/cgibin/NEISSQuery/home.aspx.
