# Supplementary material for: Infant burn injuries related to water heating for powdered infant formula preparation
Source: Front Pediatr. 2023 May 4;11:1125112. doi: 10.3389/fped.2023.1125112 (PMC10192855; doi:10.3389/fped.2023.1125112)
Supplement: Supplementary file 1 [file Table1.docx]

Supplementary Material

Infant Burn Injuries Related to Water Heating for Powdered Infant Formula Preparation

**Katelyn V. Chiang,* Erica H. Anstey, Steven A. Abrams, and Cria G. Perrine**

**Correspondence:** Katelyn V. Chiang: katelyn.chiang@emory.edu

**Supplemental Table. Example of each injury relation and cause classification – NEISS, 2017-2019**

| **Injury Relation** | **Injury Cause** | **Example** |
| --- | --- | --- |
| Related to PIF water heating | Code 1: PIF preparation | 4MOM mom mixing formula whot (*sic*) water, spilled water on abdomen; burn injury of abdomen |
| Potentially related to PIF water heating | Code 2: Hot water, use not specified | 10MOF was at home when pulled hot water onto self from pot. Dx: l side of chest scald burn; admitted |
|  | Code 3: Hot surface used to heat water, use not specified | 5MOM with burns to finger tips from touching hot kettle |
| Related to other infant feeding aspects | Code 4: Bottle warming | 9MOM in nannys (*sic*) arms at home as she got bottle out of microwave, bottle exploded and hot milk burned patient and nanny Dx 1st degree face burn/ |
|  | Code 5: Infant feeding item sanitation | 6MOF was in a carrier on dad's chest while he was boiling bottles when hot water splashed on her; 1st/2nd degree burns of right thigh and feet |
| Unrelated to infant formula or breast milk feeding | Code 6: Hot water or hot surface, used for food and beverage preparation | 4MOF being held by mom while mom was taking hot noodles out of microwave and Pt put foot in the noodles. Dx burn, foot |
|  | Code 7: Hot water or hot surface, used for bathing | 2MOF - Pt taking a bath in tub and hot water was turned on. Dx burn abdomen, burn leg |
|  | Code 8: Hot water or hot surface, used for health and beauty | 3MOM was being held by mom when she got tangled in the cord of a hot vaporizer/humidifier and hot water spilled on his right lower leg. Dx: burn of lower leg. |
|  | Code 9: Hot water or hot surface, used for household cleaning | 9MOM burned hand on hot water in humidifier; Dx burn hand |
|  | Code 10: Non-water heating appliances | 8MOF father opened oven and Pt reached up and touched oven, burn to hand. Dx: burn |
|  | Code 11: Hot liquids, use not specified | 10MOM spilled hot water or tea on his right upper thigh; 2nd degree burn of thigh |
|  | Code 12: Hot running water, use not specified | 8MOM presents with thermal burn to right foot after mother turned on water too hot. Removed right away and placed under cold water. Per grandmother, who is an RN, wounds were weeping. Dx: thermal burn |
|  | Code 13: Miscellaneous, unrelated to infant feeding | 9MOF in her pajamas. Dad holding her over the hot stove to help warm child up. No heat in house. Foot touched hot stove. Dx burn left sole |
|  | Code 14: Not enough information to determine relation to infant feeding | 12MOM spilled hot ***s (*sic*) from microwave on counter when trying to grab it. Dx: facial second degree scald burn; transferred |

Abbreviations: powdered infant formula = PIF; month old male = MOM, month old female = MOF, patient = Pt, nurse = RN, diagnosis = Dx
